# Chest Wall Reconstruction and Chemotherapy for Infectious Costochondritis Associated with Recurrent Breast Cancer

**DOI:** 10.70352/scrj.cr.25-0703

**Published:** 2026-04-01

**Authors:** Yuki Katayama, Masako Sakuragi, Mariko Matsumoto, Mitsuki Machinaga, Kasumi Ogihara, Takayo Fukuda, Satomi Shiba, Hirotoshi Kawata, Michiko Harao, Hironori Yamaguchi

**Affiliations:** 1 Department of Plastic Surgery, Jichi Medical University, Shimotsuke, Tochigi, Japan; 2 Department of Breast Oncology, Jichi Medical University, Shimotsuke, Tochigi, Japan; 3 Department of Gastrointestinal, General and Transplant Surgery, Jichi Medical University, Shimotsuke, Tochigi, Japan; 4 Department of Pathology, Jichi Medical University, Shimotsuke, Tochigi, Japan

**Keywords:** breast cancer, local neoplasm recurrence, reconstruction, costal chondritis

## Abstract

**INTRODUCTION:**

Management of chest wall recurrence after mastectomy remains debated. While surgery is invasive, both Japanese and international guidelines allow resection in selected patients and complete excision can provide durable control. With advances in systemic therapy, the value of aggressive local treatment for isolated recurrence requires reevaluation. Tumor biology and changes in receptor status after prior therapy also affect prognosis. However, infectious ribs or cartilage ulcers following chemotherapy and chest wall reconstruction are rarely reported. We describe a case of chest wall recurrence treated with multimodal therapy, including resection and reconstruction, complicated by infectious costochondritis.

**CASE PRESENTATION:**

A 55-year-old woman, first diagnosed 20 years ago with stage I breast cancer, underwent partial mastectomy and received adjuvant therapies. Sixteen years later, a new right breast mass was identified as stage IIA cancer. After chemotherapy, a total mastectomy and lymph node dissection were done. Five months of post-surgery recurrence in the right chest wall led to further chemotherapy and brachytherapy. Despite managing initial metastasis, new metastases occurred in the opposite breast and lymph nodes. During paclitaxel and bevacizumab treatment, she developed an ulcer from costochondritis, requiring surgery. Liver metastasis was later treated, maintaining an infection-free status for 6 months until her death. Effective local control allowed continuation of various chemotherapy regimens, enhancing her survival and QOL.

**CONCLUSIONS:**

In patients with chest wall recurrence complicated by refractory infection including costochondritis, surgical resection with appropriate reconstruction should be actively considered. Timely aggressive management of infection can enable further systemic therapy and may prolong survival and preserve QOL.

## Abbreviations

ER
: estrogen receptor
ESMO
: European Society for Medical Oncology
HR
: hazard ratio
ILRR
: isolated locoregional recurrence
NCCN
: National Comprehensive Cancer Network

## INTRODUCTION

The optimal management of chest wall recurrence after mastectomy remains controversial.^1–3)^ Although surgical resection is invasive, Japanese guidelines permit consideration of resection in selected patients, and several series have shown that complete excision of recurrent lesions can achieve durable local control. In addition to national guidelines, international consensus guidelines also support aggressive local treatment in select patients with isolated chest wall recurrence. The NCCN and the ESMO guidelines recommend consideration of surgery and/or radiotherapy for locoregional recurrence when durable local control is achievable and the patient’s general condition permits.^4,5)^ These recommendations underscore the importance of individualized, multidisciplinary decision-making in managing chest wall recurrence in the modern era. As systemic therapies for breast cancer continue to advance, the role and timing of aggressive local treatment for isolated chest wall recurrence require reappraisal. Tumor biology also influences outcomes after recurrence and shifts in receptor status or proliferative indices have been reported following endocrine or radiotherapy. However, complications arising in the setting of chest wall recurrence—particularly infectious rib or cartilage ulcers occurring after chemotherapy and subsequent resection with reconstruction—have not been well described. We present a case of chest wall recurrence managed with multimodal therapy, including surgical resection and chest wall reconstruction, complicated by infectious costochondritis after chemotherapy.

## CASE PRESENTATION

The case was a 55-year-old woman who underwent partial mastectomy and axillary dissection for cT1N0M0 right breast cancer (ER+, PgR+, HER2 score 1) 20 years earlier. She received adjuvant radiotherapy (right chest 66 Gy, right supraclavicular 46 Gy) and endocrine therapy (LH-RH agonist for 2 years, tamoxifen for 5 years).

Sixteen years later she developed a new right breast mass diagnosed as cT2N0M0 invasive ductal carcinoma on core biopsy (ER−, PgR−, HER2 score1). Treated as a new primary with neoadjuvant FEC (5-FU, epirubicin, cyclophosphamide) ×4 then docetaxel ×4, she achieved a partial response and underwent total mastectomy with axillary dissection. Pathology showed a 10-cm invasive ductal carcinoma (ER+, PgR+, HER2 score 1, Ki-67 54%), staged pT3N0M0 (Stage IIB).

**Fig. 1:**
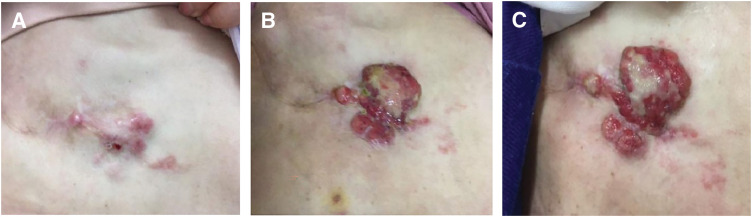
The course of chest wall recurrence lesions. (**A**) Chest wall recurrence at initiation of chemotherapy. (**B**) Treatment range for chest wall tumors defined by CT imaging. (**C**) Chest wall tumor condition before starting irradiation with needles placed.

**Fig. 2:**
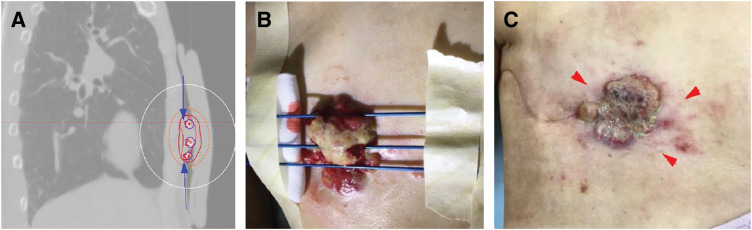
Brachytherapy delivered to the site of local chest-wall recurrence. (**A**) Treatment planning CT images for brachytherapy. (**B**) Intraoperative view of brachytherapy needle placement. (**C**) State of chest wall after 30Gy/6fr irradiations, with considerable tumor shrinkage. The tumor has shrunk within the arrowheads.

**Fig. 3:**
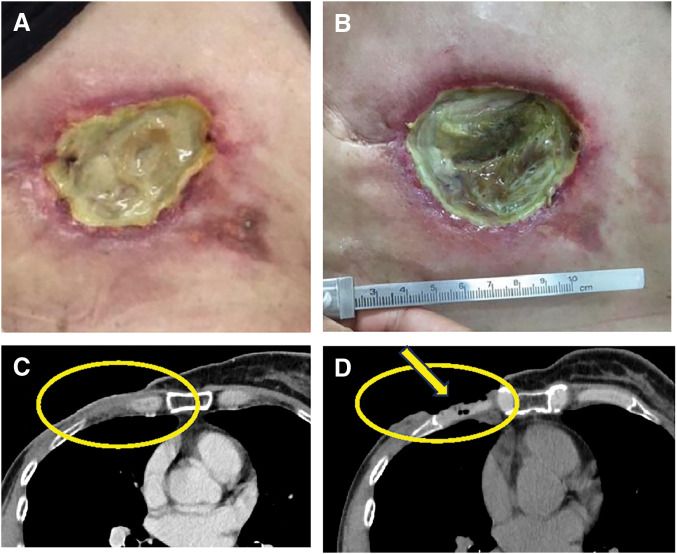
The brachytherapy-treated area became ulcerated and developed concomitant costochondritis of the ribs. (**A**, **B**) Macroscopic image (**A**) and contrast-enhanced CT (**B**) obtained shortly after treatment demonstrating marked shrinkage of the tumor mass. Reduction in ulcer depth and erythema is visible, reflecting early therapeutic response. (**B**–**D**) Late ulcerative change and costochondral destruction in the irradiated field. Gross specimen (**C**) and corresponding CT scan (**D**) showing radiation-induced ulceration at the brachytherapy site with exposure and destruction of the costal cartilage. Progressive chest wall infection and costochondritis were confirmed. The yellow circle indicates the area where the tumor has shrunk following brachytherapy. The yellow arrow points to an ulceration where air density was observed within the chest wall.

Five months after mastectomy a right chest wall tumor appeared; cytology confirmed metastatic breast carcinoma (ER−, PgR−, HER2 score 2, FISH−) (**Fig. 1**). PET-CT also showed cervical nodal recurrence without visceral metastasis. Sequential single-agent chemotherapy with eribulin, gemcitabine, and vinorelbine failed to control the expanding chest wall lesion, which was deep-seated and extensively infiltrated the chest wall. At that time, wide surgical resection was discussed in a multidisciplinary conference involving breast, plastic, and thoracic surgeons. However, surgical resection was considered unfeasible because of the depth of invasion, the anticipated extent of the chest wall defect, and concerns regarding postoperative morbidity that could compromise subsequent systemic therapy. Therefore, brachytherapy was selected as a less invasive local treatment option to achieve tumor control while preserving treatment feasibility. Brachytherapy was delivered to the chest wall lesion (5 Gy per fraction ×6 fractions, total 30 Gy), resulting in temporary local control. (**Fig. 2**). However, 1 month later a metastatic lesion appeared in the left breast with enlargement of a left axillary node; left breast core biopsy confirmed metastasis from the right primary. Combination systemic therapy with paclitaxel and bevacizumab was started but the right chest wall lesion ulcerated (**Fig. 3A** and **3B**).

**Fig. 4:**
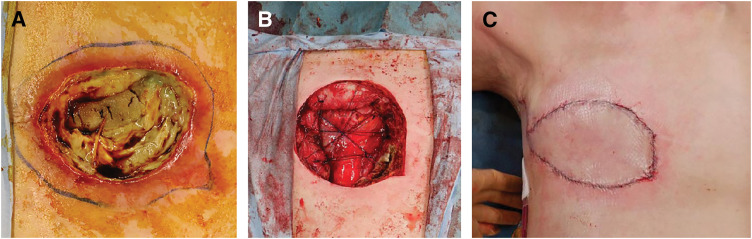
Macroscopic findings when the infected area was resected and reconstructed. (**A**) Local area ulcerated and covered with necrotic tissue before surgical resection and reconstruction. (**B**) 1-cm margin taken from erythematous area of epidermis and excision of chest wall, with bone chest wall defect shaded with 1–0 nylon. (**C**) Chest wall reconstruction using a latissimus dorsi musculocutaneous flap.

**Fig. 5:**
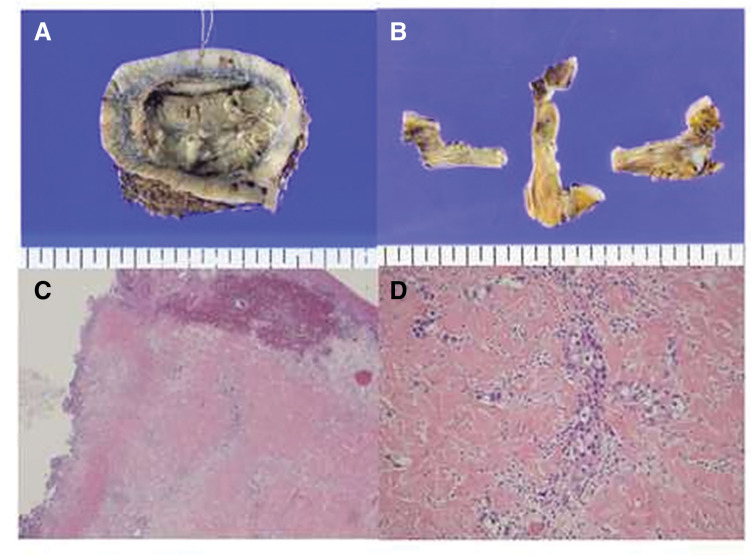
Pathologic finding of surgical specimen. (**A**) Macroscopic specimen of tumor with ulceration well-defined. (**B**) Rib cartilage at the base of the ulcer. (**C**, **D**) Microscopic findings of costochondritis at the ulcer site ((**C**)×100, (**D**)×400).

Four months after brachytherapy, the right chest wall ulcer developed infectious costochondritis with high fever (**Fig. 3C** and **3D**). Contrast-enhanced CT demonstrated gas within the rib cartilage, and culture yielded *Pseudomonas aeruginosa*. Conservative management with antibiotics failed to eradicate infection. Thoracic and plastic surgery teams performed debridement and chest wall resection, including necrotic tissue between the 4th and 6th ribs, followed by reconstruction using a pedicled latissimus dorsi musculocutaneous flap (PLDMCF). The initial closure appeared complete but postoperative fever and concern for ongoing infection prompted reoperation for staple removal, irrigation, further debridement, and re-suturing (**Fig. 4**). Superficial rib cartilage contained necrotic material with abundant bacteria. Degenerated cancer cells were present at the ulcer periphery and within the dermis, consistent with treated skin metastasis. Subtype of the surgical specimen was ER−, PgR−, HER2 score 0, Ki-67 <5%, matching the chest wall recurrence phenotype (**Fig. 5**).

**Fig. 6:**
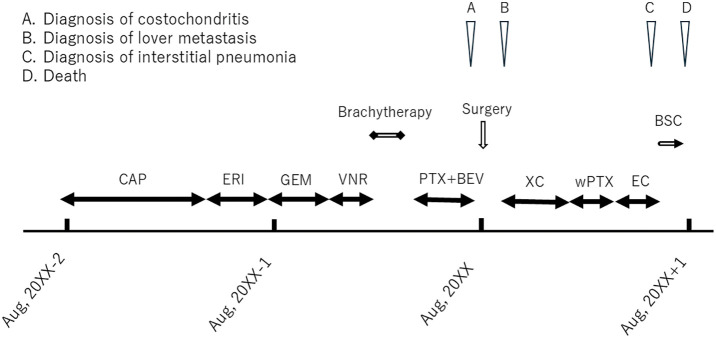
Summary of treatment progress: drugs administered and duration of chemotherapy from start of chemotherapy until death.

One month after surgery, liver metastasis was detected. As the patient’s condition improved, she received three systemic regimens over roughly 1 year (capecitabine plus cyclophosphamide; weekly paclitaxel; epirubicin plus cyclophosphamide), maintaining QOL. Thereafter she transitioned to best supportive care and died 3 months after the final chemotherapy (**Fig. 6**).

## DISCUSSION

This case describes chest-wall recurrence after resection and reconstruction complicated by infectious costochondritis. In cases with radical chest wall resection, postoperative morbidity and mortality rates were 21% and 0%, respectively, with 18% overall 5-year survival rate.^6–10)^ Wakeam et al. reported that pooled estimates from studies over the past 15 years showed a 5-year overall survival of 43.1% and a 5-year disease-free survival of 27.1% following chest-wall resection for recurrent breast cancer.^8)^

The Chemotherapy as Adjuvant for LOcally Recurrent breast cancer (CALOR) trial investigated surgical outcomes for patients with ILRR of breast cancer, comparing those who received postoperative chemotherapy with those who did not.^10)^ At a median follow-up of 9 years, there were 27 DFS events observed in the ER-negative group. The HR for a DFS event in patients with ER-negative ILRR was 0.29, corresponding to 10-year DFS rates of 70% in the chemotherapy group. Further analyses indicated HRs of 0.29 for breast cancer-free interval and 0.48 for overall survival in ER-negative patients.^11)^ Although the CALOR trial provides strong evidence supporting adjuvant chemotherapy for estrogen receptor-negative isolated locoregional recurrence, its direct applicability to the present case has limitations. Patients enrolled in CALOR predominantly had completely resected, isolated recurrences without major local complications. By contrast, our patient had unresectable chest wall recurrence complicated by prior radiotherapy, including brachytherapy, infectious costochondritis, and molecular subtype conversion. Therefore, while the CALOR trial supports the principle of systemic chemotherapy in ER-negative recurrence, extrapolation of its results to this complex clinical scenario should be interpreted with caution. Evidence supporting the use of radiation therapy for local recurrences of breast cancer is limited, with no data demonstrating that brachytherapy contributes to prolonged survival.^12)^ However, there are reports suggesting that the combination of brachytherapy with chemotherapy is well-tolerated and effective, although these conclusions are based on a small number of cases.^13)^

In the case presented here, the initial breast cancer was of luminal type, but the chest wall recurrence was triple-negative breast cancer (TNBC). Molecular subtype conversion from luminal-type primary breast cancer to triple-negative disease at recurrence is clinically significant. Such conversion is thought to reflect clonal selection under the pressure of prior endocrine therapy and chemotherapy and is associated with more aggressive tumor behavior and resistance to previous treatments. In this case, the transition to TNBC may partly explain the rapid local progression and limited response to initial systemic therapies, further supporting the need for aggressive local control. Since local control for the recurrence of the chest wall became difficult, brachy therapy was performed. Infectious costochondritis is a rare but serious complication in patients with chest wall recurrence, particularly after radiotherapy. Possible mechanisms include radiation-induced tissue necrosis, impaired vascularity of irradiated tissues, and immunosuppression related to systemic chemotherapy. Prior brachytherapy may further exacerbate local tissue damage by delivering a high radiation dose to a limited volume, predisposing cartilage and surrounding soft tissues to infection. Management typically involves prolonged antibiotic therapy; however, when infection involves necrotic cartilage or becomes refractory to conservative treatment, surgical debridement and chest wall reconstruction are often required. In the present case, infectious costochondritis represented a critical turning point that necessitated surgical intervention and enabled continuation of systemic chemotherapy. Subsequently, a skin ulcer formed in the same area with rib cartilage infection, so the patient underwent surgery with reconstruction. Thereafter, lymph node recurrence and liver metastasis occurred, but the infection was under control and chemotherapy could be administered. However, if we had not intervened with a surgical approach, the patient might have died due to infection. The surgery allowed chemotherapy to be administered for about a year following the procedure, which may have contributed to the life expectancy.^14)^

## CONCLUSIONS

In patients with chest wall recurrence complicated by refractory infection, including costochondritis, surgical resection with appropriate reconstruction should be actively considered. Timely aggressive management of infection can enable further systemic therapy and may prolong survival and preserve QOL.

## DECLARATIONS

### Availability of data and materials

The dataset for this case report is available from the corresponding author upon reasonable request.
